# Attentional Enhancement of Auditory Mismatch Responses: a DCM/MEG Study

**DOI:** 10.1093/cercor/bhu323

**Published:** 2015-01-16

**Authors:** Ryszard Auksztulewicz, Karl Friston

**Affiliations:** Wellcome Trust Centre for Neuroimaging, Institute of Neurology, University College London, London WC1N 3BG, UK

**Keywords:** attention, dynamic causal modeling, expectation, magnetoencephalography, predictive coding

## Abstract

Despite similar behavioral effects, attention and expectation influence evoked responses differently: Attention typically enhances event-related responses, whereas expectation reduces them. This dissociation has been reconciled under predictive coding, where prediction errors are weighted by precision associated with attentional modulation. Here, we tested the predictive coding account of attention and expectation using magnetoencephalography and modeling. Temporal attention and sensory expectation were orthogonally manipulated in an auditory mismatch paradigm, revealing opposing effects on evoked response amplitude. Mismatch negativity (MMN) was enhanced by attention, speaking against its supposedly pre-attentive nature. This interaction effect was modeled in a canonical microcircuit using dynamic causal modeling, comparing models with modulation of extrinsic and intrinsic connectivity at different levels of the auditory hierarchy. While MMN was explained by recursive interplay of sensory predictions and prediction errors, attention was linked to the gain of inhibitory interneurons, consistent with its modulation of sensory precision.

## Introduction

The predictive coding account of perceptual inference (Rao and Ballard 1999) entailed by the free-energy principle (Friston and Kiebel 2009; Friston 2010) has been increasingly influential in explaining how the brain uses generative models to process sensory inputs. Specifically, it has been proposed that—within cortical hierarchies—predictions about neural dynamics are continuously compared against the actual input from lower levels, and the ensuing prediction errors update the brain's generative model (Friston 2008). Previous work on the mismatch negativity (MMN)—a typical neural response to unpredicted stimuli—has suggested an underlying modulation of feedforward and feedback connectivity, implementing the propagation of sensory prediction errors and predictions, respectively (Garrido, Kilner, Kiebel, Stephan et al. 2007, 2008, Garrido, Kilner, Stephan et al. 2009; Wacongne et al. 2012).

Despite recent research, it is unclear how mismatch responses interact with top-down factors such as attention (Summerfield and Egner 2009; Lange 2013). Under predictive coding, spatial attention is characterized as contextual precision of sensory prediction errors (Feldman and Friston 2010). Accordingly, attention should increase the response amplitude to unexpected stimuli—inconsistent with the apparent consensus that MMN is pre-attentive (Näätänen et al. 2001; Garrido, Kilner, Kiebel et al. 2009). Therefore, one goal of this study was to revisit the dominant pre-attentive view of mismatch responses by replicating a few previous experiments showing a clear attentional modulation of the MMN (Woldorff et al. 1991; Sussman et al. 2013) and extending their findings to temporal attention.

More recently, predictive coding has been mapped onto distinct neuronal populations in a canonical cortical microcircuit (Bastos et al. 2012), with attention modulating the gain of superficial pyramidal cells encoding prediction errors. This has been corroborated using dynamic causal modeling (DCM) of electrophysiological data acquired in a Posner task (Brown and Friston 2013). Interestingly, however, invasive recordings in macaques suggest that attentional effects on gamma synchronization in sensory cortices (Fries et al. 2001) rely predominantly on inhibitory interneurons (Vinck et al. 2013). This distinction maps neatly onto competing explanations for gamma oscillations in cortical microcircuits, namely those maintained by recurrent interactions among inhibitory interneurons and those maintained by recurrent interactions between superficial pyramidal cells and inhibitory interneurons. Therefore, our second goal was to test the 2 accounts of attentional modulation against each other using DCM based on a canonical microcircuit (Pinotsis et al. 2012), combining the computational specificity of predictive coding schemes with a degree of neurobiological realism.

To this end, we acquired magnetoencephalographic (MEG) data in healthy volunteers in a paradigm crossing auditory expectations with temporal attention and tested for their interactive effects on event-related fields (ERFs). We modeled the underlying effective connectivity between auditory and fronto-parietal areas using DCM, hypothesizing that while mismatch processing engages a comparison of predictions and prediction errors by modulating reciprocal connections between areas (Garrido, Kilner, Kiebel, Stephan et al. 2007, 2008), attention should modulate the intrinsic gain of auditory cortices, by influencing either inhibitory interneurons (Vinck et al. 2013) or superficial pyramidal cells (Brown and Friston 2013). Our rationale was that mismatch effects are plausibly mediated by short-term plasticity in reciprocal connections due to learning of stimulus regularities, whereas attention would be mediated by contextual modulation of cortical gain.

## Materials and Methods

### Participants

Healthy volunteers (*N* = 20; 10 female; aged 19–30 years, mean ± SD: 24.57 ± 3.57 years) participated in this study upon written informed consent. Participants had normal hearing, no history of neurological or psychiatric diseases, and normal or corrected-to-normal vision. The experimental procedures were conducted in accordance with the Declaration of Helsinki (1991) and approved by the local ethics committee.

### Experimental Paradigm

**Figure 1.**
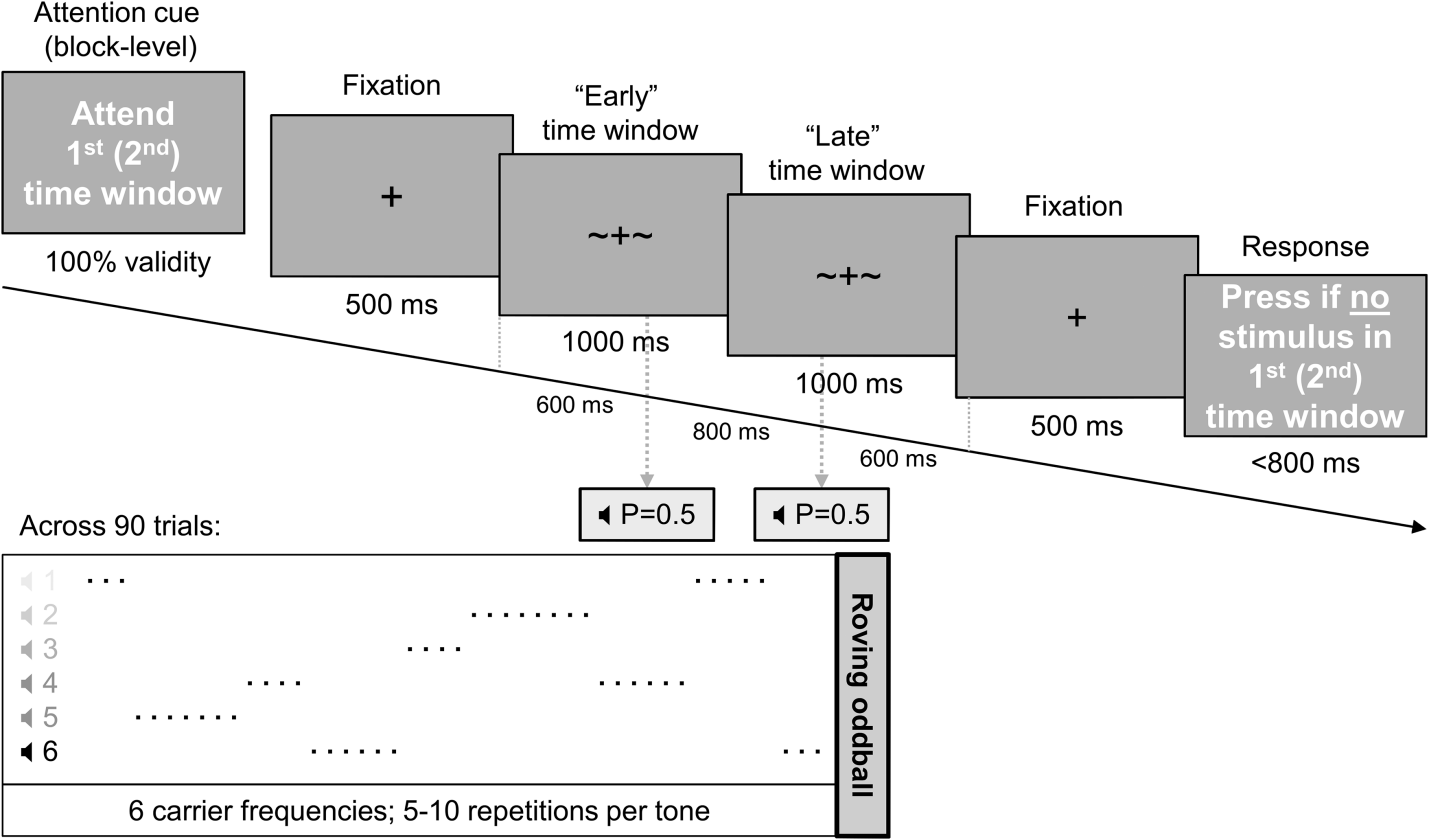
Behavioral paradigm. Auditory stimuli were presented early (600 ms after fixation offset) or late (1400 ms) in a given trial, with 50% stimulus presentation likelihood for each of the 2 latencies independently. Across trials, the stimuli formed a roving oddball sequence (panel below) of tones at 6 possible frequencies and with 5–10 repetitions per frequency. Temporal attention was manipulated at the block level, following a visual cue specifying which latency will be probed at the end of each trial for tone omission detection.

Participants performed a temporal attention task administered in 8 blocks with 90 trials in each block. In each trial (Fig. 1), after a 500-ms fixation period, auditory stimulation (consisting of 50-ms-long sine wave tones with a 20-Hz sine envelope and delivered at 6 possible carrier frequencies, between 550 and 800 Hz in steps of 50 Hz, using MEG-compatible stereo ear tubes) was presented at 2 latencies in a 2000-ms stimulation window; either 600 or 1400 ms after the offset of the fixation period. At the beginning of each block, an attentional cue specified—with 100% validity—whether participants should attend to the early or late segment in the stimulation window (randomized across blocks). In each trial, following the stimulation window and a subsequent 500-ms fixation period, participants were asked to press a button when a tone was omitted at the latency to which they were instructed to attend. Maximum response time was set at 800 ms. At each latency, tones were presented with 50% probability (independently for the 2 latencies), so that in a given trial, 0, 1, or 2 tones could be played.

Across trials, the tones formed a roving oddball sequence with 5–10 tone repetitions at each possible carrier frequency. The first occurrences of a given frequency were considered auditory deviants, and the last occurrences were defined as standards. Neural responses to the 2 types of tones were compared to reveal the effects of sensory expectation. In each block, the initial tone was discarded from the analysis.

### MEG Acquisition and Event-Related Field Analysis

MEG data were acquired using a 275-channel whole-head setup with third-order gradiometers (CTF systems) at a sampling rate of 1200 Hz. Eye movements were recorded using a non-ferrous infrared eye-tracking system (SR Research). All subsequent analyses were performed in SPM12b (Wellcome Trust Centre for Neuroimaging, University College London) for Matlab (Mathworks, Inc.).

Raw continuous MEG data were down-sampled from 1200 to 300 Hz and notch-filtered with a stop band 49–51 Hz. The vertical eye-tracker channel was used to detect eye blinks. Sensor data were corrected for eye blink artifacts by subtracting the 2 principal topography modes associated with eye blinks (Ille et al. 2002). Corrected data were epoched from −100 to +350 ms relative to auditory stimulus onset. Epoched data were baseline-corrected to the pre-stimulus period. Trials with channels containing *Z*-scored ERF amplitudes exceeding 5 standard deviations were excluded from further analysis. Data were averaged across trials using robust averaging for 4 experimental conditions: attended deviants (first tones in a given roving oddball sequence), attended standards (last tones in a sequence), unattended deviants, and unattended standards. The resulting ERF time-series were low-pass-filtered at 40 Hz.

To test for significant effects of attention and expectation on ERF amplitude, ERF time-series were converted into 3D images (topography × time) and analyzed with statistical parametric mapping using analysis of variance with 2 within-subjects factors: attention and expectation. Significant effects were inferred using random field theory (Kilner et al. 2005) after thresholding the statistical parametric maps at *P* < 0.005 (peak-level, uncorrected) and correcting *P*-values based upon cluster size for multiple comparisons using a family-wise error rate at *P* < 0.05.

### Dynamic Causal Modeling

A neural mass model based on a canonical microcircuit (Fig. 4*A*; cf. Pinotsis et al. 2012) was used for a subsequent DCM analysis, where the observed effects of experimental manipulations on ERFs are modeled as contextual changes in effective connectivity in a network comprised of several neural sources. In canonical microcircuit DCMs, the activity at each source is modeled using ordinary differential equations that describe changes in postsynaptic voltage and current in 4 neuronal populations. The 4 neural populations (spiny stellate cells in Layer 4, superficial and deep pyramidal cells in Layers 2/3 and 5/6, respectively, and inhibitory interneurons) are equipped with distinct profiles of ascending and descending connectivity both intrinsically (coupling neural populations within a source) and extrinsically (linking different sources). Specifically, spiny stellate cells in Layer 4 and deep pyramidal cells are thought to receive ascending (bottom-up) input, whereas superficial pyramidal cells and inhibitory interneurons receive descending (top-down) input. Crucially, there is a laminar asymmetry in terms of the output of each source—superficial pyramidal cells propagate signals to hierarchically higher areas (bottom-up or ascending), whereas deep pyramidal cells propagate signals to hierarchically lower areas (top-down or descending). Within sources, neural populations are interconnected with excitatory and inhibitory connections. Mathematically, the dynamics at each source are described by a set of coupled differential equations:

$$\begin{array}{ll} & {\dot{V}}_{\mathrm{SS}}=I_{\mathrm{SS}}\\ & \;\;{\dot{I}}_{\mathrm{SS}}=\;\kappa_{\mathrm{ss}}(A^{F}\sigma (V_{\mathrm{SP}})-\gamma_{\mathrm{SS}\to SS^{\sigma }}(V_{\mathrm{SS}})-\gamma_{\mathrm{SP}\to SS^{\sigma }}(V_{\mathrm{SP}})\\ & \;\;\;-\gamma_{\mathrm{II}\to SS^{\sigma }}(V_{\mathrm{II}})Cu)-2\kappa_{\mathrm{SS}}V_{\mathrm{SS}}-\kappa_{\mathrm{SS}}^{2}I_{\mathrm{SS}} \end{array}$$

$$\;\begin{array}{ll} & {\dot{V}}_{\mathrm{II}}=I_{\mathrm{II}}\\ & \;{\dot{I}}_{\mathrm{II}}=\kappa_{\mathrm{II}}(-A^{B}\sigma (V_{\mathrm{DP}})+\gamma_{\mathrm{SS}\to II^{\sigma }}(V_{\mathrm{SS}})+\gamma_{\mathrm{DP}\to II^{\sigma }}(V_{\mathrm{DP}})\\ & \;-\gamma_{\mathrm{II}\to II^{\sigma }}(V_{\mathrm{II}}))-2\kappa_{\mathrm{II}}V_{\mathrm{II}}-\kappa_{\mathrm{II}}^{2}I_{\mathrm{II}} \end{array}$$

$$\;\begin{array}{ll} & {\dot{V}}_{\mathrm{SP}}=I_{\mathrm{SP}}\\ & \;{\dot{I}}_{\mathrm{SP}}=\kappa_{\mathrm{SP}}(-A^{B}\sigma (V_{\mathrm{DP}})+\gamma_{\mathrm{SS}\to SP^{\sigma }}(V_{\mathrm{SS}})-\gamma_{\mathrm{SP}\to SP^{\sigma }}(V_{\mathrm{SP}}))\\ & \;\;-2\kappa_{\mathrm{SP}}V_{\mathrm{SP}}-\kappa_{\mathrm{SP}}^{2}I_{\mathrm{SP}} \end{array}$$

$$\begin{array}{ll} & {\dot{V}}_{\mathrm{DP}}=I_{\mathrm{DP}}\\ & \;{\dot{I}}_{\mathrm{DP}}=\kappa_{\mathrm{DP}}(A^{F}\sigma (V_{\mathrm{SP}})-\gamma_{\mathrm{DP}\to DP^{\sigma }}(V_{\mathrm{DP}})-\gamma_{\mathrm{II}\to DP^{\sigma }}(V_{\mathrm{II}}))\\ & \;\;\;-2\kappa_{\mathrm{DP}}V_{\mathrm{DP}}-\kappa_{\mathrm{DP}}^{2}I_{\mathrm{DP}} \end{array}$$

Here, the 4 neuronal populations are indicated by subscripts SS (spiny stellate cells), II (inhibitory interneurons), SP (superficial pyramidal cells), and DP (deep pyramidal cells). *V*_m_ and *I*_m_ denote the voltage and current of population *m*, with synaptic rate constant $\kappa_{m}s$. *C* is a sigmoid operator transforming the postsynaptic potential into firing rate, *A*^F^ and *A*^B^ represent the extrinsic (between regions) forward and backward connections, and *γ_m→n_* encode the intrinsic (within-region) connection from population *m* to *n*. Finally, the changes in current of spiny stellate cells at the lowest level of the hierarchy also depend on thalamic input *u* scaled by its weight *C.* This canonical microcircuit model has been used in several previous DCM studies of synaptic gain (e.g., Boly et al. 2012; Brown and Friston 2013).

Source locations were based on a multiple sparse priors source reconstruction (Friston et al. 2008) of the main effect of expectation on ERF topography at 170–230 ms post-stimulus (see Results for more details). The DCM architecture (i.e., the weighted adjacency matrix of extrinsic connections among sources) was optimized using fixed-effects Bayesian model selection following a heuristic model search: First, the basic architecture was identified using responses to “unattended standards.” Changes in extrinsic connectivity were then selected under this basic architecture using responses in all conditions. Finally, expectation and attention-dependent changes in intrinsic connectivity were identified. In all 3 steps, models were inverted using a 1- to 300-ms peristimulus time window, which included both main effects of attention and expectation and their interaction. The thalamic input to A1 was modeled as a Gaussian function with a prior latency of 20 ms post-stimulus. The DCMs were completed with a spatial forward model (mapping from source dipoles to observed MEG topography) based on a single MEG shell (Nolte 2003).

The first step considered 9 competing model structures, differing in the number of sources and in the pattern of extrinsic connections (Fig. 4*B*). The 9 models were inverted per participant to model the “unattended standard” ERFs. These responses were considered the baseline for subsequent modulation by attention and expectation. The selected model structure was then optimized with respect to condition-specific changes in extrinsic connectivity. Sixteen competing models, each allowing for a different subset of connections (forward, backward, both, or no connections) to be modulated by either of the experimental factors (attention and/or expectation), were fitted to each participant's ERF data and compared using fixed-effects Bayesian model selection based on the free-energy approximation to their log-evidence (Friston et al. 2007). This approach implements the a priori assumption that each participant's data were generated under the same (unknown) model—and ensures that models are compared based on a tradeoff between their accuracy and complexity (Stephan et al. 2009). Finally, the model with an optimized modulation of extrinsic connections was used to compare alternative models of intrinsic modulation by attention and expectation.

The canonical microcircuit neural mass model has been considered in terms of the message passing implicit in predictive coding (Bastos et al. 2012). Crucially, the precision of prediction errors pertaining to hidden causes (that link levels of hierarchical models) and states (that link dynamics over time within one level) have been associated with the gain of superficial pyramidal cells and inhibitory interneurons, respectively (Feldman and Friston 2010; Friston 2010). Given the literature explaining both attention and sensory learning in terms of precision of prediction errors and the underlying synaptic gain (Brown and Friston 2013; Moran et al. 2013), the alternative models of intrinsic modulation by attention and/or expectation allowed for activity-dependent gain modulation of either superficial pyramidal cells or inhibitory interneurons at different levels of the processing hierarchy, resulting in 7 models per experimental factor. As mentioned above, the models were compared based on their free-energy approximation to log model evidence using a fixed-effects Bayesian model selection. The winning model was used to infer the posterior connectivity and gain parameters after Bayesian parameter averaging (Garrido, Kilner, Kiebel, Friston et al. 2007).

## Results

### Behavioral Results

Temporal attention and sensory expectation were orthogonally manipulated in an auditory mismatch paradigm (Fig. 1; see Methods) where participants were instructed to detect tone omissions at 1 of 2 latencies. Participants (*N* = 20) correctly detected tone omissions in mean ± SD of 84.02 ± 11.50% of the trials. The detection rates were marginally different between attentional conditions (two-sample *t*-test, *P* = 0.055) and did not differ between deviants and standards (*P* = 0.12). Data from 2 participants—whose performance was at chance in single blocks toward the end of the run—were discarded from further ERF analysis.

### Event-Related Fields—Sensor Space Analysis

**Figure 2.**
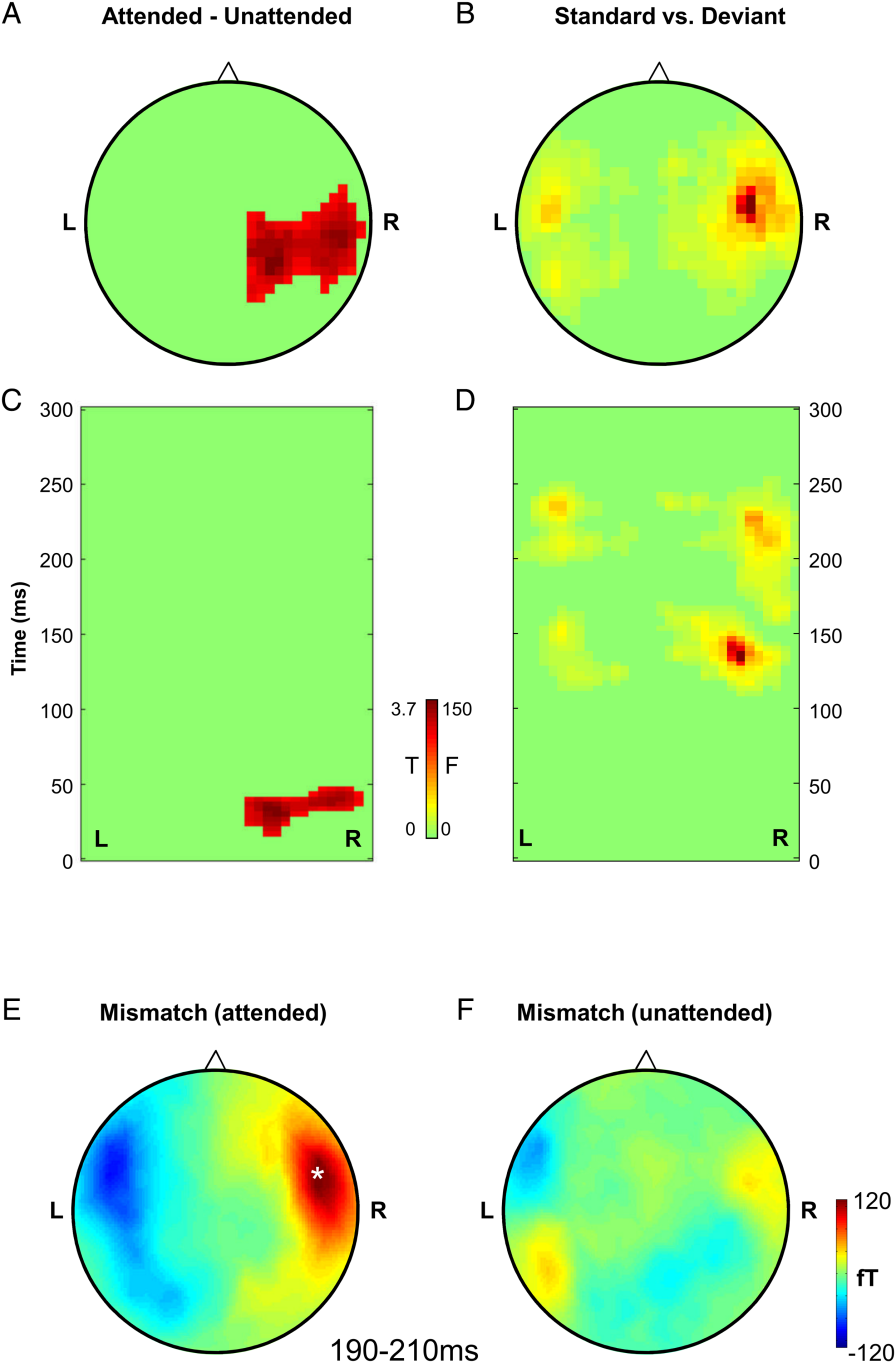
Effects of attention (top row) and expectation (bottom row) on ERF amplitude. (*A*–*D*) Left column: the topography of significant effects; the main effect of attention thresholded at *T* = 2.68 (*P* < 0.005 peak-level, corrected for multiple comparisons at a cluster-level p_FWE_ < 0.05); the main effect of expectation thresholded at *F* = 10.34 (*P* < 0.005 peak-level, corrected for multiple comparisons at a cluster-level p_FWE_ < 0.05). Right column: the timing of the significant effects (same thresholding as for the topography plots; *x*-axis: left-right topography, *y*-axis : peristimulus time). (*E*,*F*) Topography of the mismatch response (auditory standards vs. deviants) for the attended (left) and unattended (right) conditions. Plots show ERF amplitude averaged over 190–210 ms post-stimulus, corresponding to the timing of a significant interaction between attention and expectation (*P* < 0.005 peak-level, corrected for multiple comparisons at a cluster-level p_FWE_ < 0.05). Asterisk indicates the topography of the significant interaction cluster. Post-hoc paired *t*-tests revealed that ERF amplitude over right fronto-temporal channels was significantly different between standards and deviants for the attended condition, but not for the unattended condition.

To rule out possible confounds due to motor preparation, only trials in which auditory tones were presented at both early and late latencies were analyzed. After artifact rejection, an average (over subjects) of 19.28 trials (SD 3.97) was used in the “attended deviant” condition, 17.61 trials (SD 3.20) in the “attended standard” condition, 19.28 trials (SD 3.21) in the “unattended deviant” condition, and 16.28 trials (SD 4.24) in the “unattended standard” condition. Individual participants’ ERFs were entered into an analysis of variance with 2 factors: attention and expectation. The main effects and interactions were based on cluster size (over an uncorrected threshold of *P* < 0.005) and corrected for multiple comparisons using a family-wise error rate *P* < 0.05. Attention had an effect on ERFs as early as 27–40 ms post-stimulus, over right centro-temporal channels (attended vs. unattended stimuli; peak-level *T*_max_ = 4.40; cluster-level p_FWE_ = 0.046; Fig. 2*A*,*C*).

Expectation violation (deviants vs. standards) had an effect on 2 subsequent ERF components, an earlier component at 123–143 ms post-stimulus (peak-level *T*_max_ = 3.92; cluster-level p_FWE_ = 0.037; left centro-parietal channels) and later at 170–237 ms (peak-level *T*_max_ = 6.33; cluster-level p_FWE_ < 0.001; right fronto-temporal channels). The opposite contrast (standards vs. deviants) revealed significant differences in ERF amplitude at 127–170 ms (peak-level *T*_max_ = 4.95; cluster-level p_FWE_ < 0.001; right centro-parietal channels) and 210–233 ms (peak-level *T*_max_ = 5.08; cluster-level p_FWE_ = 0.001; left fronto-temporal channels). Since the polarity of evoked responses differed across hemispheres (cf. Fig. 2*E*,*F*), the main effect of expectation is depicted in Figure 2*B* and *D*, based on an F-contrast of deviants vs. standards. This disclosed 2 significant components—an earlier component at 110–163 ms post-stimulus (right centro-parietal channels: peak-level *F*_max_ = 159.89, cluster-level p_FWE_ < 0.001; left centro-parietal channels: 120–150 ms post-stimulus, peak-level *F*_max_ = 34.17; cluster-level p_FWE_ < 0.001) and later at 163–240 ms (right fronto-temporal channels: peak-level *F*_max_ = 70.19, cluster-level p_FWE_ < 0.001; left fronto-temporal channels: 210–233 ms, *F*_max_ = 49.80, cluster-level p_FWE_ < 0.001; Fig. 2*B*,*D*). Therefore, the topography and timing of effects based on unidirectional and bidirectional contrasts were largely identical.

Crucially, there was a significant interaction between attention and expectation at 193–197 ms (peak-level *T*_max_ = 4.56; cluster-level p_FWE_ = 0.009). Post-hoc paired *t*-tests revealed that while there was a significant mismatch response (deviants vs. standards) in the attended condition (190–210 ms, peak-level *T*_max_ = 7.24; cluster-level p_FWE_ < 0.001; right fronto-temporal channels), expected and unexpected tones did not differ significantly in the unattended condition (all cluster-level p_FWE_ ≥ 0.2; Fig. 2*E*,*F*). In other words, the interaction reflected an effect of expectation that was only seen under attention.

### Dynamic Causal Modeling—Source Space Analysis

**Figure 3.**
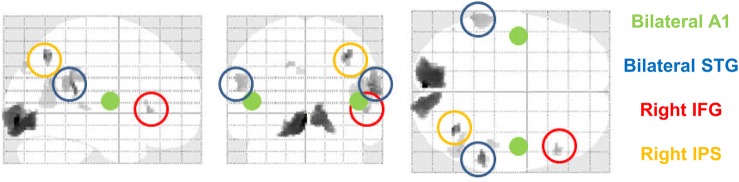
Source selection for DCM. Network nodes were selected based on a multiple sparse priors source reconstruction of the mismatch response (deviants vs. standards) using a time window in which there was a significant interaction between attention and expectation. Sources in STG, the right IFG, and the right intraparietal sulcus (IPS) were used to model the observed effects. Additionally, sources in bilateral primary auditory cortices (A1) were included in all models. See main text for details.

The prior location of the cortical sources included in subsequent dynamic causal models was based on a source reconstruction of ERFs corresponding to the 4 conditions (attended standards, attended deviants, unattended standards, and unattended deviants) at 170–237 ms post-stimulus (the time window of a significant main expectation effect on ERP magnitude, including the time window of a significant interaction between attention and expectation). Following multiple sparse priors source reconstruction (Friston et al. 2008), condition-specific responses (evoked power on the cortical surface) were analyzed using analysis of variance as mentioned above. Statistical parametric maps were inspected at an omnibus threshold of *P* < 0.05 (uncorrected) to identify candidate neural sources of the effects observed on ERF amplitude. When comparing deviants vs. standards (Fig. 3), sources in bilateral superior temporal gyri (STG; MNI coordinates: left [−60, −48, 20], right [56, −40, 18]) and the right inferior frontal gyrus (IFG; MNI coordinates: [52, 24, 0]) were identified and included in subsequent DCMs—as in previous DCM of the MMN (Garrido, Kilner, Kiebel, Stephan et al. 2007, 2008). Furthermore, the right inferior parietal sulcus (IPS; MNI coordinates: [34, −66, 46]) was included, because it has been implicated in explicit timing (Coull and Nobre 2008). Finally, sources in bilateral primary auditory cortices (A1) were added, given their plausible involvement in processing the auditory stimuli. The prior location coordinates for the A1 sources were taken from previous modeling work of the MMN (Garrido et al. 2008; right A1: MNI [46, −14, 8], left: MNI [−42, −22, 7]). Since the primary goal of the current analysis was to model the effects of attention and expectation in auditory cortical microcircuitry, other candidate sources (e.g., in visual areas)— possibly engaged during the processing of visual cues—were not included in subsequent DCMs.

**Figure 4.**
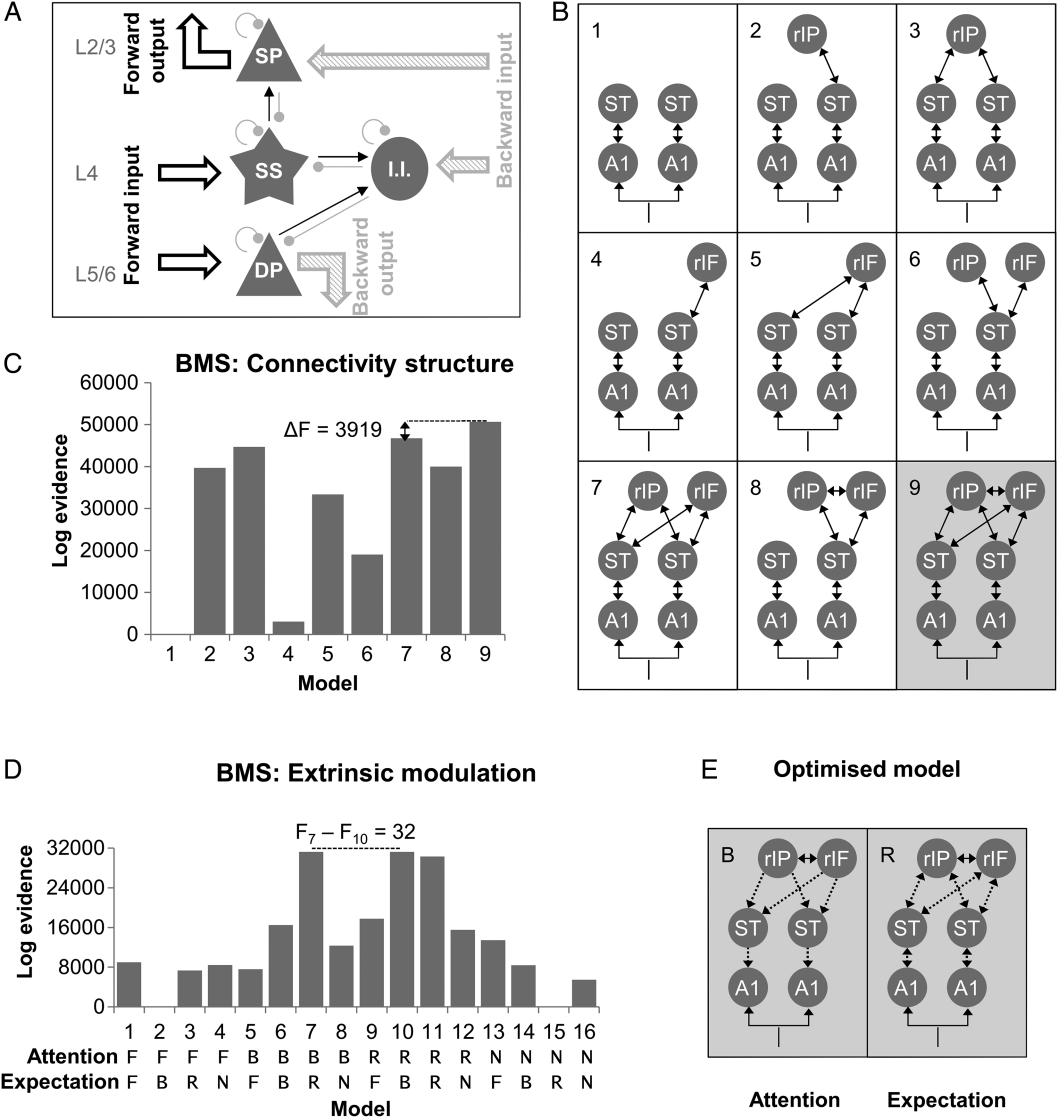
Dynamic causal modeling: optimizing the extrinsic connectivity structure. (*A*) All DCMs were based on a canonical microcircuit source architecture. Each source is modeled using 4 neuronal populations (spiny stellate cells in Layer 4, superficial and deep pyramidal cells in Layers 2/3 and 4/5, respectively, and inhibitory interneurons), linked by ordinary differential equations describing their current and voltage dynamics, and differing with respect to their intrinsic connectivity (with other populations; thin arrows, black: excitatory, red: inhibitory) and extrinsic connectivity (with other sources; thick arrows). The ascending extrinsic connections are considered excitatory and represent prediction errors, whereas the descending extrinsic connections are considered inhibitory and represent sensory predictions. Finally, each population is characterized by a gain parameter (inhibitory self-connections) encoding precision. (*B*) 9 alternative models were fitted to individual subjects' ERFs corresponding to the unattended auditory standards. All models included thalamic auditory input to bilateral A1 and differed with respect to the number of fronto-parietal sources and the extrinsic connectivity between them and the rest of the network. (*C*) Fixed-effects Bayesian model selection revealed that the model (shaded gray in the left panel) including both fronto-parietal sources (rIF: right inferior frontal gyrus; rIP: right intraparietal sulcus) and bilateral connectivity with the superior temporal gyrus sources (ST) outperformed all other models. (*D*) Modeling the contextual effects on extrinsic connectivity. 16 alternative models were designed, where each contextual factor (i.e., attention and expectation) could modulate a different subset of extrinsic connections between bilateral A1 and STG and between bilateral STG and the fronto-parietal sources: only feedforward connections (models “F”), only feedback connections (models “B”), both feedforward and feedback connections (models “R”), or no extrinsic connections (models “N”). Models were compared using fixed-effects Bayesian model selection. (*E*) The winning model had a posterior probability of >99% and allowed for both forward and backward connections to be modulated by expectation, but only the feedback connections to be modulated by attention.

Following the selection of candidate sources, model structure was optimized by comparing 9 alternative models (Fig. 4*B*) of responses to unattended standards (using a fixed-effects Bayesian model comparison). This procedure indicated that the model with both the right IFG and IPS sources connected to each other—as well as bilaterally to the STG sources—outperformed competing models (Fig. 4*C*; difference in log model evidence [i.e., log Bayes factor] to the second-best model: 3919, indicating very strong evidence in favor of the winning model; cf. Penny et al. 2004).

The selected model was used to further optimize condition-specific changes in extrinsic connectivity. In this step, 16 alternative models were compared; with each condition-specific effect (attention and expectation) modulating a different subset of extrinsic connections (only forward, only backward, forward and backward, or null models with no modulation). These models were fitted to each subject's data to explain observed differences in ERF amplitude. A fixed-effects Bayesian model selection revealed that the model with (i) attentional modulation of backward connections and (ii) a modulation of both forward and backward connections by expectation outperformed all other models (Fig. 4*D*,*E*; log-evidence difference compared with the second-best model: 32, corresponding to a Bayes factor of exp(32), or >99% posterior confidence in the winning model). An additional analysis including models that allowed for a modulation of extrinsic connections by the interaction of attention and expectation (replacing the main effects) showed that, on average, models with interaction effects had less evidence than the models based on main effects. This finding was largely expected because the (gain) effects of attention and expectation in the DCM are highly nonlinear and can easily explain interactions in source space.

**Figure 5.**
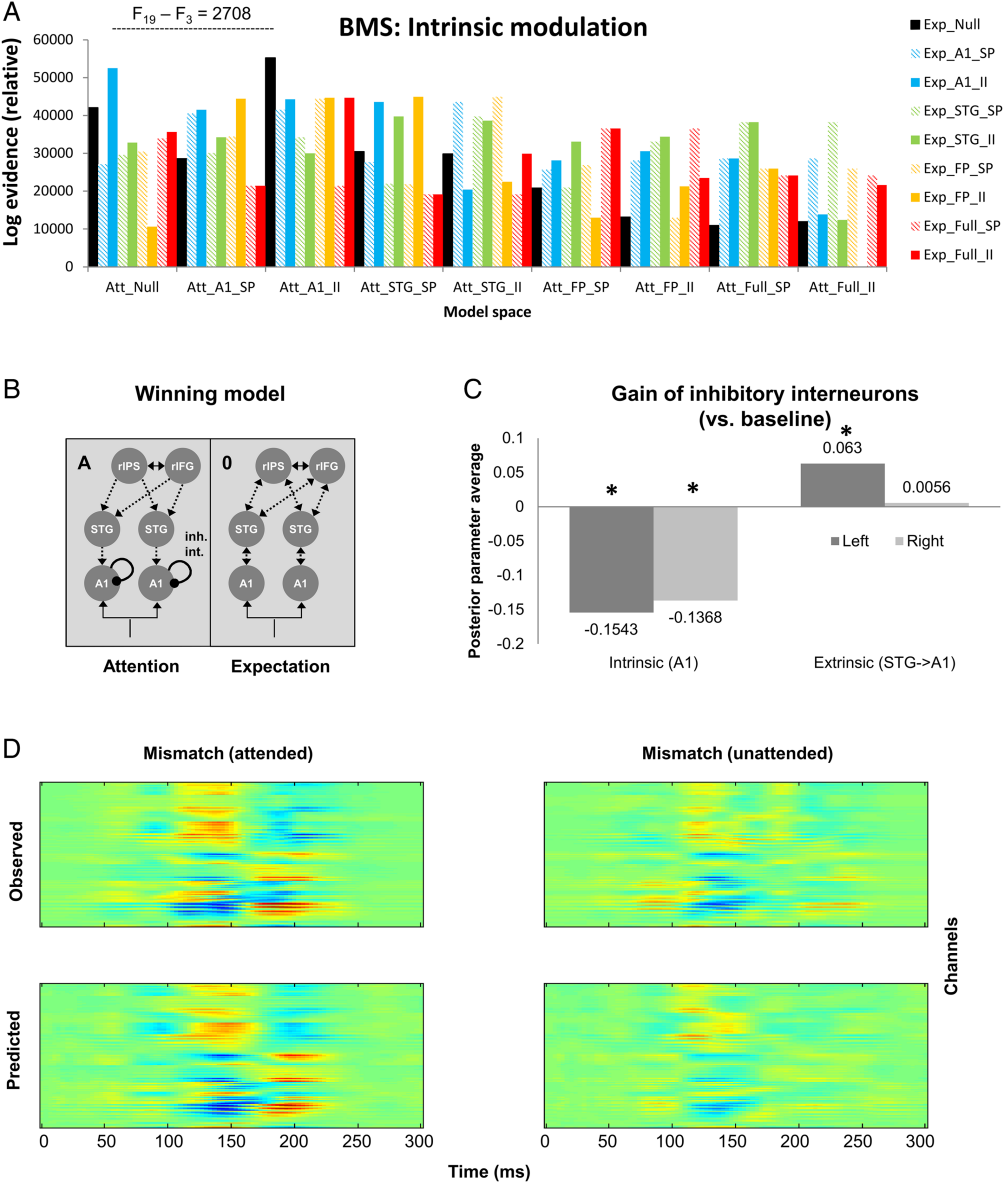
Modeling the contextual effects on intrinsic connectivity. (*A*) Each contextual factor could modulate a different subset of intrinsic connectivity parameters. The null models were equivalent to the winning model in Figure 4*E*, allowing for only extrinsic connectivity modulation by attention or expectation (models labeled “Null”). In further models, intrinsic modulation by attention (Att) and/or expectation (Exp) was placed in bilateral A1 on either the superficial pyramidal cells (“A1_SP”) or inhibitory interneurons (“A1_II”), in bilateral STG (superficial pyramidal cells: “STG_SP,” inhibitory interneurons: “STG_II”), in the fronto-parietal sources (superficial pyramidal cells: “FP_SP,” inhibitory interneurons: “FP_II”), or at all 3 hierarchical stages (superficial pyramidal cells: “Full_SP,” inhibitory interneurons: “Full_II”). (*B*) The winning model allowed for an attentional modulation of the gain of inhibitory interneurons in bilateral A1. (*C*) Posterior mean of parameters encoding the change of activity-dependent gain of inhibitory interneurons due to attention (relative to the unattended baseline; left panel) and the attention-dependent modulation of the extrinsic top-down inhibitory connection from STG to A1. For both left and right A1 sources, the gain of inhibitory interneurons is significantly stronger following attention (>99% posterior probability). The top-down connection is significantly modulated only in the left hemisphere. (*D*) Model fits of the winning model. Top row: observed responses over 275 MEG channels and 0–300 ms post-stimulus time. Bottom row: responses predicted by the winning model. Columns correspond to mismatch responses for attended and unattended conditions, respectively.

Finally, to test whether attention and expectation modulate the gain of specific neuronal populations, 81 competing models were designed, whereby attention and expectation could modulate intrinsic gain of either superficial pyramidal cells or inhibitory interneurons, over different levels of the hierarchy (A1, STG, or fronto-parietal sources). A set of null models with no intrinsic modulation, as well as a set of full models with intrinsic modulation at all hierarchical levels, were considered. As before, alternative models were fitted to individual subjects’ data and compared using a fixed-effects Bayesian model selection (Fig. 5*A*; log-evidence difference between the 2 highest-scoring models: 2708, indicating >99% posterior confidence in the winning model).

The winning model included an attentional modulation of the gain of A1 inhibitory interneurons and extrinsic backward connectivity strength, whereas unexpected stimuli modulated both forward and backward extrinsic connectivity (Fig. 5*B*). Quantitative estimates of effective connectivity and their modulation were averaged across participants using Bayesian parameter averaging (over subjects) under this winning model. The gain of inhibitory interneurons in both left and right A1 was significantly stronger under attention (posterior probability of a significant decrease in self-inhibition >99% in both left and right A1; Fig. 5*C*). As the gain of inhibitory interneurons was modeled as activity dependent (i.e., scaled by the input from higher areas), the winning model allowed for a modulation of intrinsic gain by backward (descending) extrinsic afferents. Because the gain of inhibitory interneurons is mediated by inhibitory recurrent or self-connections, attentional modulation appears to be consistent with a top-down disinhibition of intrinsic neuronal activity that is mediated by inhibitory interneurons. Figure 5*C* also shows attentional modulation of the STG->A1 top-down (inhibitory) connection, which was only significant in the left hemisphere. The winning model showed an excellent correspondence of predicted and observed data for all MEG channels and time points used in the inversion (Fig. 5*D*).

**Figure 6.**
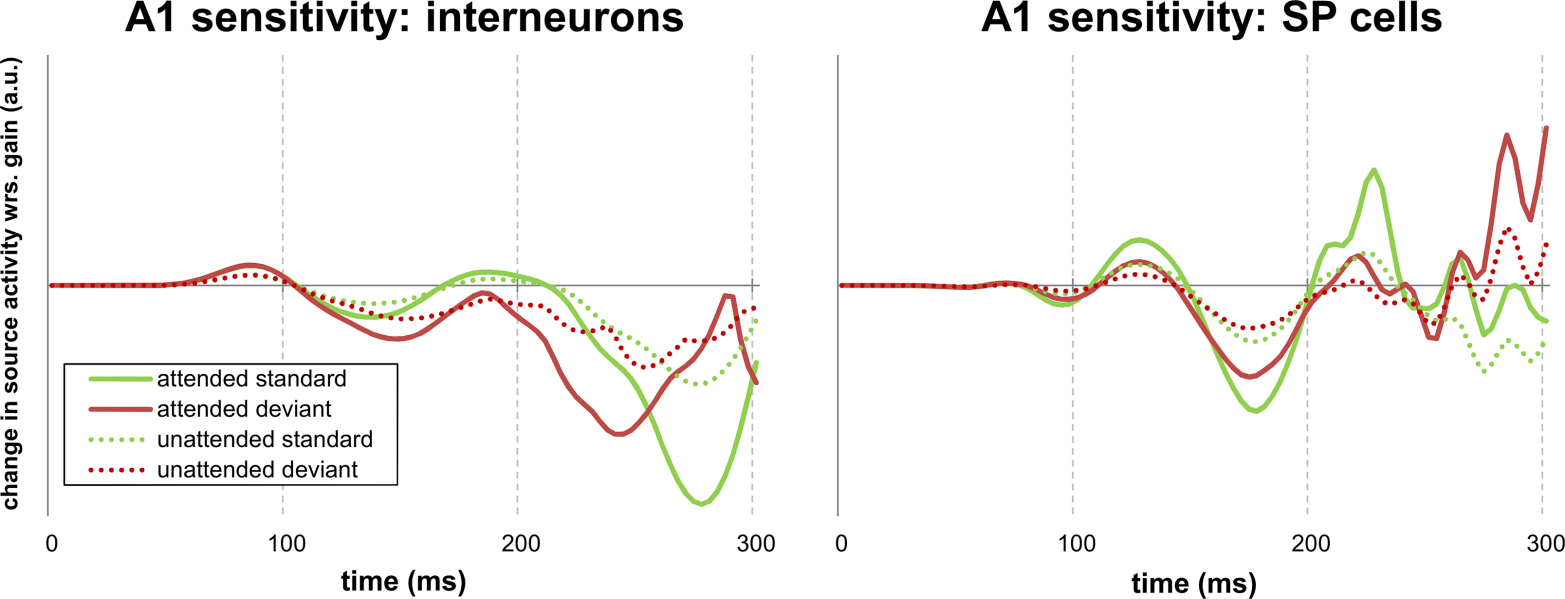
Contribution analysis. Changes in A1 source activity as a function of changes in state-dependent gain of inhibitory interneurons (left panel) and superficial pyramidal cells (right panel), averaged across hemispheres. Group posteriors of parameters were obtained from fixed-effects Bayesian parameter averaging across subjects. Gain modulation of inhibitory interneurons leads to an earlier differential response between attended and unattended stimuli, as compared with gain modulation of superficial pyramidal cells.

To illustrate how changing specific parameters affects source activity, we performed a contribution analysis of the gain of inhibitory interneurons in bilateral A1 (Fig. 6). To this end, we used averaged posterior parameter estimates of 2 models—the winning model, in which attention modulated the gain of inhibitory interneurons, and a competing model with attentional gain modulations in the superficial pyramidal cell population. These group posteriors were based on fixed-effects Bayesian parameter averaging across subjects. We then assessed how increasing the state-dependent gain of inhibitory interneurons vs. superficial pyramidal cells would affect source activity in A1 (averaged across hemispheres). As depicted in Figure 6, gain modulation of inhibitory interneurons leads to an earlier differential response between attended and unattended stimuli than gain modulation of superficial pyramidal cells. This is in accordance with the early onset of the main effect of attention observed in our data. Furthermore, changes in gain of inhibitory interneurons are associated with temporally smoother effects on A1 source activity than changes in gain of superficial pyramidal cells, consistent with the effects seen in Figure 2*D*. Note that these contribution analyses are consistent with an increase in gain through a disinhibition of inhibitory neurons (i.e., reduced sensitivity in the right panel of Figure 6 with increasing self-inhibition). Furthermore, these sensitivity profiles illustrate nicely how interactions in sensor space (between attention and expectation) can be explained by separable but nonlinear (gain) effects of attention and expectation at the neuronal level—as hypothesized under predictive coding schemes.

## Discussion

We modeled MEG data acquired in a task combining temporal attention and an auditory roving oddball paradigm to disentangle the neural mechanisms of attention and expectation. Mismatch responses to frequency deviants were strongly modulated by temporal attention, speaking against the common interpretation of the MMN response as being pre-attentive (Näätänen et al. 2001; Garrido, Kilner, Kiebel et al. 2009). Instead, our results are in line with a few studies showing a modulation of the MMN by attention (Woldorff et al. 1991; Sussman et al. 2013) and, furthermore, allow for an extrapolation of these earlier findings to the domain of temporal attention.

More generally, the observed interaction of attention and sensory predictions is entirely consistent with the predictive coding account of attention in perceptual inference. Under predictive coding, sensory inputs are continuously compared against the predictions entailed by a generative model (Rao and Ballard 1999; Friston 2010), which itself is optimized by the ensuing prediction errors. Crucially, the predictive coding framework formalizes attention as (Bayes) optimal updating of sensory precision (Feldman and Friston 2010), whereby more precise sensory prediction errors are manifest as stronger evoked responses that lead to larger updates at higher levels of the generative model. It has been previously established that casting attention as the optimization of sensory precision offers a mechanistic explanation for both neural and behavioral effects typically seen in a visuospatial Posner task (Feldman and Friston 2010; Brown and Friston 2013). In those studies, attention was modeled as input-dependent precision which 1) modulates the response of a particular neuronal population (e.g., pyramidal cells or inhibitory interneurons) to presynaptic inputs and 2) depends on the conditional expectations from higher hierarchical levels. This corresponds to a top-down control of synaptic gain and is consistent with the modulatory effects of top-down cortico-cortical connections (Bastos et al. 2012). Here, instead of spatial attention, we have manipulated temporal attention to interleaved stimuli presented over the course of many trials. Although previous work on temporal orienting has focused on its pre-stimulus correlates—in terms of the phase of ongoing low-frequency oscillations in sensory cortex (Lakatos et al. 2008; Arnal and Giraud 2012)—early evoked responses are typically enhanced by experimental manipulations of temporal attention based on the task relevance of stimuli (Lange 2013). Attentional boosting of evoked responses is consistent both with the early effects of attention observed in the current data set as well as with previous modeling work on spatial attention (Feldman and Friston 2010).

Furthermore, unlike in the Posner paradigm (Posner 1980), we have manipulated attention in a filtering rather than probabilistic fashion (Lange 2013). Specifically, to ensure that attention and sensory expectations were orthogonal to each other, 1) the attentional cue indicated which time window would be probed at the end of the trial with 100% validity, 2) the stimulus identity (i.e., the auditory frequency) was irrelevant for the tone omission task, and 3) attending to a given time window was not predictive of the likelihood of a stimulus being presented in this time window. The relatively low difficulty of the task might have attenuated the behavioral effects of attention (see Results). However, the principal aim of the current paradigm was to manipulate temporal attention (i.e., the relevance of stimulus timing for the task at hand) independently of stimulus presentation likelihood, which ensured that temporal attention was not confounded with contextual expectancies of stimuli occurring at a particular latency. The relatively early onset of the observed attentional effects on ERF amplitude (27–40 ms) is consistent with previous results obtained in attentional paradigms based on auditory filtering (Rif et al. 1991) and can be interpreted as direct evidence of attentional gating (Lange 2013).

The relation of attention to other contextual factors is subject to an ongoing debate. Several recent papers have addressed the interaction of attention and expectation, suggesting either their synergistic (Hsu et al. 2014) or antagonistic (Kok et al. 2012) effects. In the current experiment, we aimed to manipulate first-order sensory predictions, where auditory deviants violate the sensory predictions established by preceding tone repetitions. Using a similar approach in the visual modality, fMRI studies of repetition suppression have brought evidence for a dependence of repetition-induced expectation effects on both spatial attention (Eger et al. 2004; Henson and Mouchlianitis 2007) and feature-based attention (Yi and Chun 2005; Yi et al. 2006; Moore et al. 2013). Extending these previous findings to temporal auditory attention, the interactive effects of attention (sensory precision) and expectation (the difference between predictions and incoming sensory input) reported here can be therefore explained in terms of attentional scaling of prediction errors. In contrast to manipulating first-order sensory predictions by, for example, stimulus repetition, some of the previous experimental manipulations of expectation have been contextual in nature, where a particular stimulus can be more or less expected (anticipated) in a given setting due to its occurrence frequency (e.g., Larsson and Smith 2012; Jiang et al. 2013), associative content (Chaumon et al. 2013) or regularity within a stimulus stream (Hsu et al. 2014). In predictive coding schemes, manipulating second-order (contextual) expectancies of stimuli would be equivalent to increasing the precision of prediction errors higher in the processing hierarchy and therefore have a positive (modulatory) effect on stimulus-evoked responses, similar to—and synergistic—with the effects of attention (Larsson and Smith 2012; Hsu et al. 2014).

Both in the canonical microcircuit for predictive coding (Bastos et al. 2012) and in previous modeling work on attention (Feldman and Friston 2010; Brown and Friston 2013) and sensory precision (Brown and Friston 2012), precision has been associated with gain of superficial pyramidal cells, which are thought to implement the comparison of (descending) sensory predictions from higher levels of the hierarchy with (ascending) input from the lower levels of the hierarchy and propagate the ensuing prediction errors (pertaining to hidden causes) forward along the processing stream. The current DCM analysis suggests, however, that temporal attention modulates the gain of inhibitory interneurons—in addition to modulating the strength of top-down inhibitory connections across the network. This model outperformed the model allowing for a direct modulation of the gain of superficial pyramidal cells. Having said this, the gain of superficial pyramidal cells is usually modeled in terms of a reduction in inhibitory self- or recurrent connectivity that, implicitly, implicates inhibitory interneurons. In predictive coding, inhibitory interneurons have been linked to signaling prediction errors on the hidden states (which model the conditional dependencies over time; cf. Friston 2008; Bastos et al. 2012). Our modeling results are consistent with a recent neurophysiological study in macaques (Vinck et al. 2013), where the attentional synchronization of single-unit spiking activity to the local field potentials in the gamma frequency band has been shown to primarily rely on the activity of inhibitory interneurons and not pyramidal cells, suggesting a predominant role of inhibitory interneurons in generating cortical gamma and setting synchronous gain (Chawla et al. 1999).

**Figure 7.**
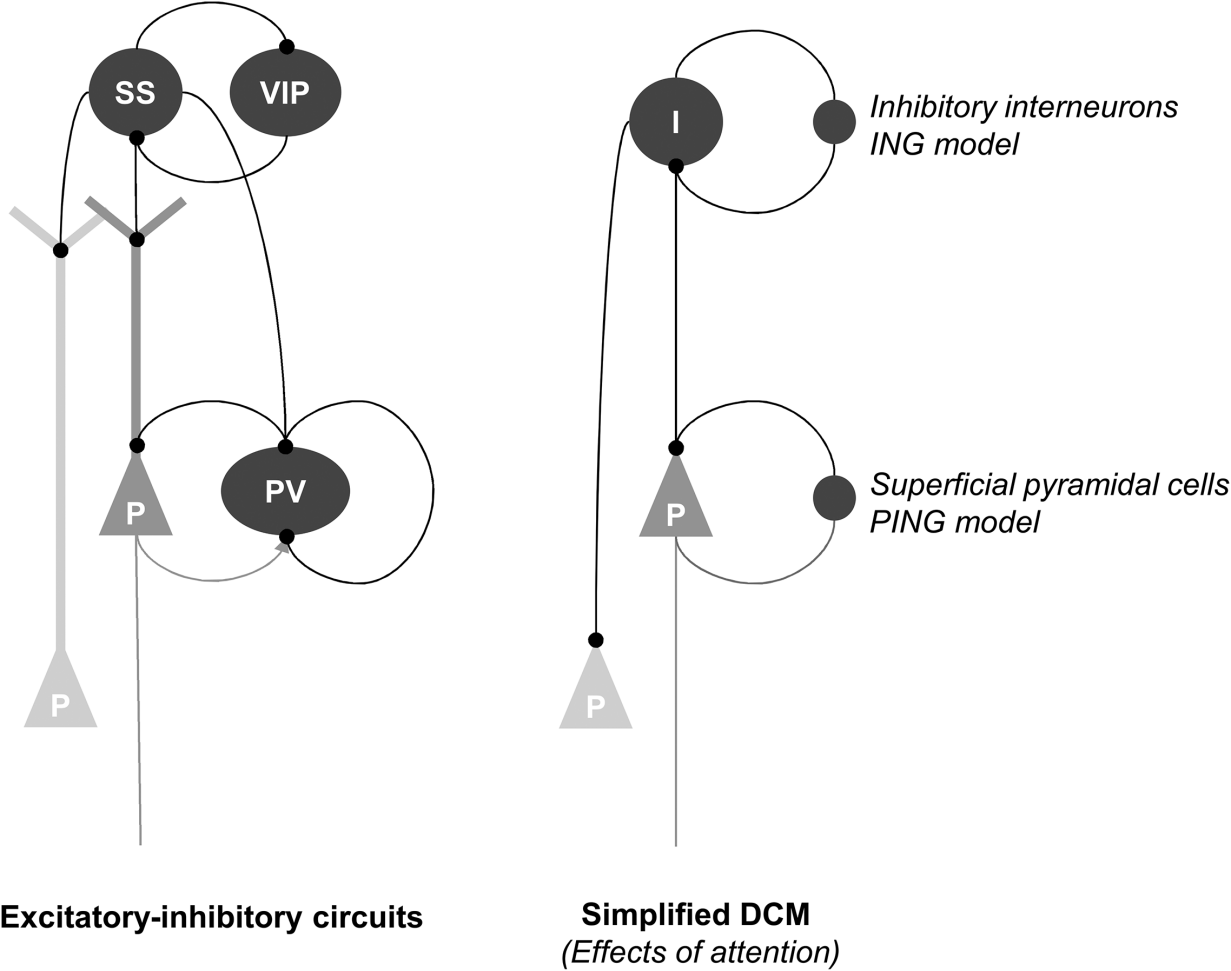
The left panel depicts interactions between (superficial and deep) pyramidal cells with inhibitory interneurons. We have divided the inhibitory interneurons into 3 dominant subtypes (Parvalbumin-positive PV, somatostatin SST, and vasoactive intestinal peptide expressing interneurons, VIP). The intrinsic connectivity is based upon the recent optogenetic studies (Pfeffer et al. 2013), nuanced to fit our purposes. In brief, we have assumed that PV interneurons are densely and reciprocally connected to the pyramidal cells, particularly through perisomatic compartments, whereas SST cells form synapses on their dendrites. The right panel shows a simplified architecture implicit in our dynamic causal models. Here, we have absorbed the recurrent inhibitory (PV/pyramidal cell) dynamics into an inhibitory recurrent connection, whereas the SST/VIP interneurons provide (dendritic) inhibitory drive. This allows us to map the ING and PING models onto the canonical microcircuits used in DCM. In this setting, the PING model emphasizes recurrent interactions among PV cells as modeled by the inhibitory recurrent connections on superficial pyramidal cells. In contrast, the ING model corresponds to the influence of (SST/VIP) inhibitory interneurons on pyramidal cells.

Formally speaking, there are 2 competing explanations for the genesis of gamma activity in local microcircuits. The first inhibitory interneuron network gamma (ING) model supposes that excitatory pyramidal cells are entrained by recurrent interactions among inhibitory neurons. Conversely, the pyramidal cell interneuron network gamma (PING) model calls upon reciprocal message passing between pyramidal and inhibitory interneurons to maintain fast gamma activity. Our DCM results appear to support the ING perspective; if we allow for a simple mapping between changing the gain of recurrent (inhibitory) connections on superficial pyramidal cells and inhibitory cells with the PING and ING models, respectively: See Figure 7. Having said this, DCM does include reciprocal intrinsic connections between superficial pyramidal cells and inhibitory interneurons. The oscillatory mechanisms underlying the attentional gain modulation of inhibitory interneurons are clearly an important focus for future work.

In summary, we have demonstrated that mismatch responses can be explained in terms of changes in extrinsic connectivity mediating sensory predictions and prediction errors, reflecting short-term plasticity associated with the learning of stimulus regularities. Crucially, the sensory prediction errors are modulated by their precision following temporal attention. Neurophysiologically, the attentional gain modulation might predominantly rely on the neuromodulation of inhibitory interneurons. Our modeling results support the predictive coding account of perceptual inference, where precise inhibitory interneuron signaling should result in more efficient updating of the hidden states describing the temporal dynamics of the generative model. This provides a biologically plausible mechanistic explanation of the interactions between top-down perceptual effects and sensory processing in terms of hierarchical message passing in cortical circuits.

## Funding

This work was funded by the Wellcome Trust and the German Research Foundation. The Wellcome Trust Centre for Neuroimaging is supported by core funding from the Wellcome Trust 091593/Z/10/Z. Funding to pay the Open Access publication charges for this article was provided by the Wellcome Trust.
